# Arnold-Chiari Malformation Type II and CYP1B1 Congenital Glaucoma: A Possible Association

**DOI:** 10.1155/2021/4808346

**Published:** 2021-09-21

**Authors:** Shaikha Aldossari, Amani Al Bakri, Yumna Kamal

**Affiliations:** ^1^King Khaled Eye Specialist Hospital, Riyadh, Saudi Arabia; ^2^King Abdulaziz University, Jeddah, Saudi Arabia

## Abstract

**Background:**

We describe a case of an infant with Arnold-Chiari Malformation Type II (ACM-II) who was born with lumbosacral myelomeningocele, hydrocephalus, and primary congenital glaucoma (PCG) together with dysmorphic features (scaphocephaly, frontal bossing, hypotelorism, entropion, and flat nasal bridge), which according to our knowledge, is a combination that has yet to be described in literature. *Primary diagnosis.* A 2-year-old female who is known to have ACM-II was referred due to abnormal eye examination done in a peripheral hospital that suggested infantile glaucoma in both eyes. *Findings.* During her last physical exam (postop), she was vitally stable, conscious with good feeding. Ophthalmic assessment revealed buphthalmia, superior paracentral scar, deep anterior chambers (AC), and round pupils with positive red reflex, clear lens, and an IOP of 16, 14 mm Hg, respectively. Neurological exam showed paraparesis and moving upper extremities and has axial hypotonia. Genetic testing showed CYP1B1 gene mutation.

**Conclusion:**

The aim of reporting this case is to share the findings in this infant as it may be a new association. The main learning message here is that ACM-II patients may present with certain ocular symptoms, including glaucoma-related ones that may mimic neurological disorders. This report brings information that could alert general practitioners, neurologists, and neurosurgeons. A deeper understanding of this rare disorder may aid the diagnosis of cases with similar characteristic physical findings by referring them to an ophthalmology clinic for further evaluation. *Case presentation.* A 2-year-old female who is known to have Arnold Chiari Malformation Type II (ACM- II) was referred due to abnormal eye examination done in a peripheral hospital that suggested infantile glaucoma in both eyes. MRI at 3 months of age showed lumbosacral myelomeningocele and hydrocephalus. Genetic testing confirmed a CYP1B1 mutation. These combinations of symptoms were never described in the literature before.

## 1. Introduction

Chiari malformations are a result of multifactorial etiology, mainly genetic and environmental ones. They are classified based on their morphology and severity of anatomic defects. Chiari I malformation is characterized by abnormally shaped cerebellar tonsils that are displaced below the level of the foramen magnum. Type II, also known as Arnold Chiari Malformation (ACM), is when both the cerebellum and brainstem pass through foramen magnum in the presence of spina bifida. This type has been reported to co-occur with known genetic syndromes involving but not limited to trisomy 18 [1]. Primary pediatric glaucomas are subdivided into many subtypes, one of which is primary congenital glaucoma (PCG) which is defined as the presence of isolated abnormal trabecular meshwork (TM) during first three years of life [2]. It is inherited in an autosomal recessive pattern. Due to the high rate of consanguinity in Saudi Arabia, PCG was found to be highly prevalent, especially those caused by CYP1B1 mutations [3]. The two main genes linked to PCG are cytochrome P450 1B1 gene (CYP1B1) within GLC3A locus and LTBP2 within GLC3C locus. The only two ophthalmic findings that were reported in the literature to be associated with Arnold Chiari Malformation Type II are abnormal ocular motility with impairment of smooth pursuit [4, 5].

## 2. Case Report

A 2-year-old female who is known to have Arnold Chiari Malformation Type II was referred due to abnormal eye examination done in a peripheral hospital that suggested infantile glaucoma in both eyes for which she was put on topical Xolamol and Travatan.

She was born to a healthy 18-year-old woman who had an uncomplicated pregnancy course apart from oligohydramnios at 35 weeks' gestation (amniotic fluid index: 7.7 cm, deepest pool 4.3 cm). Mother denied taking folic acid supplementation before or during first trimester. The patient is a product of full-term cesarean section due to breach presentation and large head size. Good APGAR score 6 and 8 at 1 and 5 minutes, respectively, admitted to NICU for myelomeningocele and hydrocephalus. No history of seizures, developmental delays, or neurological disorders in her family. MRI at 3 months of age showed lumbosacral myelomeningocele and hydrocephalus. Ventriculoperitoneal (VP) shunt was inserted at 2 weeks of age and followed by repair of dorsolumbar myelomeningocele at 3 months of age.

During her last physical exam (postop), she was vitally stable, conscious with good feeding. Ophthalmic assessment revealed buphthalmia, superior paracentral scar, deep anterior chambers (AC), and round pupils with positive red reflex and clear lens, and IOP was 16, 14 mm Hg, respectively. Neurological exam showed paraparesis, moving upper extremities, and has axial hypotonia. Skin evaluation revealed large scar of surgery in the posterior dorsolumbar area from repaired meningomyelocele.

The rest of her physical examination was unremarkable. Patient was in good general condition without any distress. She had abnormally shaped head (scaphocephaly). Anterior fontanel was open and flat. VP shunt present. Tympanic membranes and throat were clear. There was no lymphadenopathy. Chest was clear to auscultation, and no rhonchi or crepitations were heard.

On examination, the patiemt had regular heart rate and rhythm without murmurs, a soft and non-tender abdomen, no organomegaly, and normal extremities.

Axial eye length (AEL) is as follows: OD 24.50 mm and OS 25.18 mm. Lab results showed relative neutropenia of 23% (normal: 40–74%). Genetic testing confirmed CYP1B1 mutation.

Deep sclerectomy (DS) was done at 5 months of age (December 2018) for both eyes at different times, one week apart. Micropulse CPC OD was done at the age of 19 months (February 2020). 11 days post-DS, she developed bilateral shallow choroidal detachment with macular involvement from 7.30 to 10.30 extending from disc to almost anterior equator in the right eye and from 1.30 to 4.30 extending from disc to almost anterior equator (except superior temporally to posterior equator only) in the left eye. Plan was to examine her under sedation in two weeks, and she was put on Pred Forte (prednisolone acetate ophthalmic suspension every 4 hours and Atropine twice daily OU.

One-month post-DS, IOP was digitally high and unmeasurable by the Tonopen due to high reading, and pupil was mid-dilated. B-scan showed bilateral optic disc cupping worse in the left eye and resolution of choroidal detachment. This could either indicate that she is a steroid responder or surgery failure due to dilation. Plan was to taper PF, stop atropine, and start antiglaucoma medication.

Despite adequate compliance to full topical antiglaucoma medications, IOP readings remained continuously high upon follow up in the right eye. At age 29 months (December 2020), patient underwent surgery to insert Aurolab aqueous drainage implant (AAIDI Valve) OD.

Post-AAIDI, the patient was doing well. IOP improved but remained high, patient continued Xolamol OD and Travatan.

The rest of her clinical course was unremarkable to date.

## 3. Discussion

Arnold Chiari Malformation Type II (ACM-II) has many etiologies, one of which is of genetic bases as it was linked to certain genetic syndromes 1. Primary congenital glaucoma occurs most commonly in a sporadic fashion, and only about 10-40% were found to be familial and of autosomal dominant inheritance pattern [6].

In our patient, genetic testing confirmed a cytochrome P4501B1 (CYP1B1) gene mutation, which is present on GLC3A loci. This gene was the first gene to be reported as a cause of primary congenital glaucoma [7, 8].

These two entities might have a possible association since both have genetic bases. A study found that 97 out of 126 Arnold Chiari Malformation patients had ocular disturbances [9], mainly nystagmus, since 70% of Arnold Chiari Malformation patients had it in another study [10], specifically, downbeat nystagmus [11].

When it comes to type II, the only ophthalmic finding that was reported in the literature to be associated with Arnold Chiari Malformation Type II was abnormal ocular motility with impairment of smooth pursuit [4, 5].

However, for type I, previous studies reveal a wider range of clinical findings that involved decreased visual acuity in both eyes [12], restricted eye movement [12], rotational nystagmus [12], megalocornea [12], stage II bilateral papilledema [12], synechial angle-closure glaucoma [13], and acquired esotropia [14].

Although a single case reported glaucoma development in a patient with (ACM-I) [15], another one denied the presence of goniodysgenesis or high IOP in a patient with the same type of malformation [12].

To date, the effect of Arnold Chiari Malformation Type II on iridocorneal angle structures was not described in the literature before, whether these two entities are related to each other or it was a coincidence is still in question.

## Additional Points

*Literature Search*. Databases searched were mainly PubMed and Google Scholar, and search terms used were Arnold-Chiari Malformation Type II (ACM-II), primary congenital glaucoma (PCG), dysmorphic features, and syndrome. Years covered were from 1977 to 2020.

## References

[1] Tubbs R. S., Hill M., Loukas M., Shoja M. M., Oakes W. J. (2008). Volumetric analysis of the posterior cranial fossa in a family with four generations of the Chiari malformation type I. *Journal of Neurosurgery. Pediatrics*.

[2] Badeeb O. B. (2011). *Congenital Glaucoma in Saudi Arabia*.

[3] Badeeb O. M., Micheal S., Koenekoop R. K., den Hollander A. I., Hedrawi M. T. (2014). CYP1B1 mutations in patients with primary congenital glaucoma from Saudi Arabia. *BMC Medical Genetics*.

[4] Salman M. S., Dennis M., Sharpe J. A. (2009). The cerebellar dysplasia of Chiari II malformation as revealed by eye movements. *The Canadian Journal of Neurological Sciences*.

[5] Salman M. S., Sharpe J. A., Lillakas L., Steinbach M. J., Dennis M. (2007). Smooth ocular pursuit in Chiari type II malformation. *Developmental Medicine & Child Neurology*.

[6] Lewis C. J., Hedberg-Buenz A., DeLuca A. P., Stone E. M., Alward W. L. M., Fingert J. H. (2017). Primary congenital and developmental glaucomas. *Human Molecular Genetics*.

[7] Weinreb R. N., Grajewski A. L., Papadopoulos M., Grigg J., Freedman S. F. (2013). *Childhood glaucoma: The 9th Consensus Report of the World Glaucoma Association*.

[8] Bejjani B. A., Xu L., Armstrong D., Lupski J. R., Reneker L. W. (2002). Expression Patterns of Cytochrome P4501B1 (*Cyp1b1*) in FVB/N Mouse Eyes. *Experimental Eye Research*.

[9] Milhorat T. H., Chou M. W., Trinidad E. M. (1999). Chiari I malformation Redefined: clinical and radiographic findings for 364 symptomatic patients. *Neurosurgery*.

[10] Dobkin B. H. (1977). The adult Chiari malformation. *Bulletin of the Los Angeles Neurological Societies*.

[11] Menezes A. H., Smoker W. R. K., Dyste G. N., Youmans J. R. (1990). Syringomyelia, Chiari malformations, and hydromyelia, chapter 46. *Neurological Surgery*.

[12] Imane M., Asmae M., Toufik R., Rachid S. (2016). Œdème papillaire revelant une malformation d’Arnold Chiari type 1: à propos d’un cas. *The Pan African Medical Journal*.

[13] Dagi L. R., Walton D. S. (2006). Anterior Axial Lens Subluxation, Progressive Myopia, and Angle-Closure Glaucoma: Recognition and Treatment of Atypical Presentation of Ectopia Lentis. *Journal of American Association for Pediatric Ophthalmology and Strabismus*.

[14] Lewis A. R., Kline L. B., Sharpe J. A. (1996). Acquired esotropia due to Arnold-Chiari I malformation. *Journal of Neuro-Ophthalmology*.

[15] Schijman E. (2004). History, anatomic forms, and pathogenesis of Chiari I malformations. *Child's Nervous System*.

